# Workload Assessment of Tractor Operations with Ergonomic Transducers and Machine Learning Techniques

**DOI:** 10.3390/s23031408

**Published:** 2023-01-27

**Authors:** Smrutilipi Hota, V. K. Tewari, Abhilash K. Chandel

**Affiliations:** 1 Agricultural and Food Engineering Department, Indian Institute of Technology Kharagpur, Kharagpur 721302, WB, India; 2 Department of Biological Systems Engineering, Virginia Tech Tidewater AREC, Suffolk, VA 23437, USA; 3 Center for Advanced Innovation in Agriculture (CAIA), Virginia Tech, Blacksburg, VA 23437, USA

**Keywords:** tractors, dynamic operator workload, lower limb muscles, ergonomic transducers, machine learning

## Abstract

Dynamic muscular workload assessments of tractor operators are rarely studied or documented, which is critical to improving their performance efficiency and safety. A study was conducted to assess and model dynamic load on muscles, physiological variations, and discomfort of the tractor operators arriving from the repeated clutch and brake operations using wearable non-invasive ergonomic transducers and data-run techniques. Nineteen licensed tractor operators operated three different tractor types of varying power ranges at three operating speeds (4–5 km/h), and on two common operating surfaces (tarmacadam and farm roads). During these operations, ergonomic transducers were utilized to capture the load on foot muscles (gastrocnemius right [GR] and soleus right [SR] for brake operation and gastrocnemius left [GL], and soleus left [SL] for clutch operation) using electromyography (EMG). Forces exerted by the feet during brake and clutch operations were measured using a custom-developed foot transducer. During the process, heart rate (HR) and oxygen consumption rates (OCR) were also measured using HR monitor and K4b2 systems, and energy expenditure rate (EER) was determined using empirical equation. Post-tractor operation cycle, an overall discomfort rating (ODR) for that operation was manually recorded on a 10-point psychophysical scale. EMG-based maximum volumetric contraction (%MVC) measurements revealed higher strain on GR (%MVC = 43%), GL (%MVC = 38%), and SR (%MVC = 41%) muscles which in normal conditions should be below 30%. The clutch and brake actuation forces were recorded in the ranges of 90–312 N and 105–332 N, respectively and were significantly affected by the operating speed, tractor type, and operating surface (*p* < 0.05). EERs of the operators were measured in the moderate-heavy to heavy ranges (9–24 kJ/min) during the course of trials, suggesting the need to refine existing clutch and brake system designs. Average operator ODR responses indicated 7.8% operations in light, 48.5% in light-moderate, 25.2% in moderate, 10.7% in moderate-high, and 4.9% operations in high discomfort categories. When evaluated for the possibility of minimizing the number of transducers for physical workload assessment, EER showed moderate-high correlations with the EMG signals (***r***_GR_ = 0.78, ***r***_GL_ = 0.75, ***r***_SR_ = 0.68, ***r***_SL_ = 0.66). Similarly, actuation forces had higher correlations with EMG signals for all the selected muscles (***r*** = 0.70–0.87), suggesting the use of simpler transducers for effective operator workload assessment. As a means to minimize subjectivity in ODR responses, machine learning algorithms, including K-nearest neighbor (KNN), random forest classifier (RFC), and support vector machine (SVM), predicted the ODR using body mass index (BMI), HR, EER, and EMG at high accuracies of 87–97%, with RFC being the most accurate. Such high-throughput and data-run ergonomic evaluations can be instrumental in reconsidering workplace designs and better fits for end-users in terms of agricultural tractors and machinery systems.

## 1. Introduction

The safety and efficiency of agricultural machinery operators have been ever-increasing global concerns. This requires reductions of the involved risks and muscle fatigue through appropriate workplace design or refinements based on operator workload assessments [1,2,3,4]. Electromyography (EMG) is one of the latest and widely used technology for dynamic muscle fatigue and activity assessment [4,5]. EMG is a composite of all the muscle fiber action potentials occurring in the muscles beneath the skin [6,7]. EMG captures small electrical signals called electromyograms as the result of ion exchange across muscle fiber membranes prior to force generation from muscle contractions. EMG is measured by placing electrodes on the skin surface (non-invasive, surface EMG [sEMG]) or inserted in the muscle (invasive, intramuscular EMG [iEMG]) [8,9]. sEMG is commonly used due to its non-invasiveness, minimal risk involvement, and ease of frequent use [8,10]. Typical EMG features utilized for muscle activity assessments include root mean square (RMS), mean frequency (MNF), median frequency (MDF) and percentage of maximum voluntary contraction known as muscle workload (%MVC) [4]. %MVC is obtained as the ratio of the RMS signal for the muscle under contraction in real time to the signal for muscle under maximum contraction measured without any constraints or load, which represents muscular involvements for a given operation [11]. EMG also enables the identification of comfort level and body postures for a given operation [12,13].

In the year 2000, EMG was used to evaluate muscle fatigue during repeated isokinetic knee extension, i.e., under peak muscle torque, and strong positive correlations were observed between MNF and peak torque [14]. For the human elbow muscles, the effect of joint angle has also been evaluated through relationships between applied relative force and MVC [15]. It was observed that the EMG amplitude for the biceps, brachioradialis and triceps muscles was better determined by the required percentage of available force with the muscles compared to the absolute required force. A similar study identified the Gaussian and positive linear relationship between applied force and EMG signals for bicep muscles as feasibility to understanding occupational fatigue or neuromuscular disorders [16]. A meta-study conducted using globally published data observed EMG as a better characterizer compared to traditional approaches such as Rapid Entire Body Assessment (REBA), Rapid Upper Limb Assessment (RULA), Ovako working posture assessment system (OWAS), and psychophysical scales, such as body parts discomfort scale (BPDS), and overall discomfort rating (ODR). The study also observed EMG to be a very widely adopted approach for the real-time and high-throughput monitoring of muscle fatigue and musculoskeletal risks in laboratories and industries of production engineering [4].

Among the use cases, EMG was utilized to identify muscle activity redundancy and fatigue during pedal operation in passenger cars and identified that as the pedal stroke increases, the muscle activation increases [17]. The EMG was further used to evaluate the fatigue behavior of forearm muscles during power gripping by racing motorcycle riders [18]. The posture and braking operations were simulated and a significant positive relationship was observed between the normalized MVC and the duration of the task when 5 s contractions were interspersed at 30% of maximum muscle contraction by 5 s of recovery. At the same time, no relationship was observed when 10 s contractions at 50% of maximum muscle contraction were interspersed by 1 s recovery. The findings suggested short recovery periods (5–10 s) after maximum muscle contraction for the healthy operation of motorbikes [18].

In the aviation sector, EMG has been used to evaluate the back-muscle fatigue of pilots as a result of vibration and postural redundancies [19]. A similar study evaluated the neck stress of jet pilots through the fatigue of the left and right sternocleidomastoid, upper trapezius and middle trapezius muscles using EMG, and significant sensitivities were observed. Study findings suggested that helmet and seat designs be reconsidered [20]. EMG has also been used recently for autonomous steering control of passenger cars, and a high path with the following accuracy was obtained [21]. Most of the fatigue evaluation research has been limited to passenger vehicles or aircraft, but the load and muscular fatigues of tractors or agricultural machinery operators are rarely recognized.

Agricultural mechanization and automation have increased significantly in recent decades, where intensified field operations are carried out using tractors or tractor-driven machinery systems. However, the study of the safety and ergonomics of tractor operators has not improved and requires critical attention [13]. Tractor or tractor-driven machinery operates in tough and rugged conditions and therefore requires highly variable and redundant actuation forces compared to other automobiles [22,23]. Operators are also subjected to prolonged and redundant vibration and shock exposures at high frequencies and magnitudes that can be hazardous to musculoskeletal disorders (MSDs), lower back pain and other cardiovascular problems [13,23,24,25]. Such problems are further complemented by inappropriate workspace designs, limited ergonomic upgradations, awkward operating postures, prolonged working hours, repeated body movements, and over-exertions that eventually risk work-time loss, increased costs, and injuries [26,27,28]. As discussed and demonstrated by the previous studies on passenger vehicles, motorbikes, and aircraft, muscle fatigue and workload assessments can aid workplace design reconsiderations and safe ergonomics in mechanized agriculture.

Traditionally, tractor operator workload assessments have been conducted using REBA, RULA, OWAS, BPDS, and ODR-based scales and methods and using physiological measurements of working heart rate (HR), oxygen consumption rate (OCR), energy expenditure rate (EER) and blood pressure (BP) [25,29,30]. These studies provided overall assessments of operator health and level of exertions during operation but did not quantify the muscular fatigue or loads. EMG was recently deployed to identify comfortable operator hand positions during tractor operations [13]. The elbow angle of 100° and inclination of the steering column against the horizontal plane of 50° was identified as the most comfortable setting for upper limbs. The operators who operated a non-power-steering tractor encountered the highest load.

A recent, similar study in the US Midwest evaluated the activity levels of upper body muscle groups; erector spinae, upper trapezius, forearm flexor, and forearm extensor, using EMG for general agriculture and machinery operations [31]. Muscle fatigue was significantly affected by the equipment, tools, and work practices where the upper arm postures and movement speeds did not confer excessive risk to the shoulders. Although these studies provided reasonable evaluations for upper body muscles, sufficient studies on workload assessments of lower limbs and pertaining muscles during tractor operations are still lacking. A study from Iran was conducted in 2016 where aggregate pain thresholds of four lower limb muscles, gastrocnemius, trapezius and quadrate’s lumborum were quantified using algometers during the clutch operation of two different tractors [32]. The study observed the highest pain threshold reduction for the quadratus lumborum muscle and clutching forces to be above the allowable limit for both tractors. In the end, clutch design modifications were suggested. This was just one study that utilized a force transducer to quantify the aggregate pain threshold for lower limb muscles. Similar studies are required for a range of operating conditions, tractor or tractor-run machinery types, and geographical regions. Most importantly, in this domain, high-throughput ergonomic sensors and data-run techniques that provide continuous data in real-time and enable comprehensive analysis of lower limb muscle workload, fatigue, and discomfort are still not explored and addressing this gap is our goal.

This study contributes to the larger goal by focusing on the tractors and tractor operators in Indian conditions. Specifically, we aim to (1) quantify dynamic muscle fatigue on lower limb muscles, foot workload, and overall physiological responses using EMG, ergonomic force transducer, and biomechanical transducers during tractor clutch and brake operations in the field and normal road conditions, (2) we will also evaluate the possibility of limiting the number of transducers to quantify muscle-specific activity by investigating relationships between force and energy requirements, and dynamic load on the muscles, and (3) finally, we aim to develop a non-subjective classification of overall discomfort from transducer responses using machine learning algorithms.

## 2. Materials and Methods

### 2.1. Tractors and Operator Selection

The study was conducted in the year 2021. Three tractors in power ranges of 47–60 HP were selected from the farm shed of the Agricultural and Food Engineering Department, Indian Institute of Technology, Kharagpur, India. These tractors included a New Holland 6010, John Deere 5055E, and Escort 355 (Details in Table 1).

Nineteen licensed tractor operators with more than five years of driving experience and no prior reported musculoskeletal disorders or injuries were selected. Their average age ranged between 35.6 ± 3.5 years, weight between 72 ± 2.6 kg, height between 171 ± 2 cm, and body mass index (BMI) between 24.4 ± 0.6 kg/m^2^. Appropriate consent for participation in the study was also collected prior to the evaluation trials.

### 2.2. Data Collection

**Figure 1:**
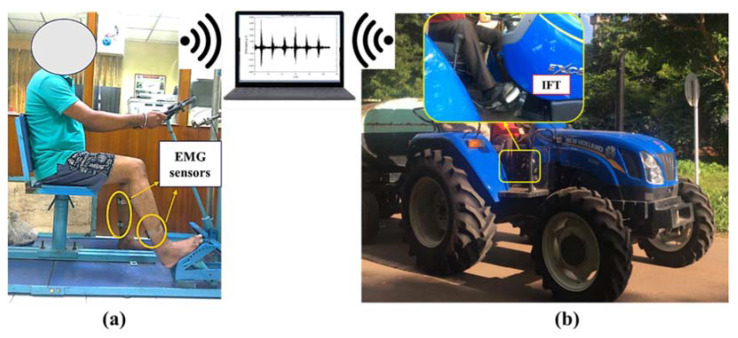
Operator electromyography and load measurement on (**a**) strength measurement setup, and (**b**) during tractor clutch and brake operation.

Tractor clutch and brake operations involve frequent increases and decreases of knee and ankle angles and engage gastrocnemius and soleus muscles of the lower limbs [32]. The gastrocnemius is a calf surface muscle that flexes the knee and foot. The soleus is a broad muscle in the lower calf, used for plantar flexion (downward foot movement away from the body) and dorsiflexion (foot’s backward bending or contraction). Therefore, gastrocnemius right (GR), soleus right (SR) and gastrocnemius left (GL), and soleus left (SL) muscles were identified for fatigue evaluations. Above the skin over these muscles, EMG sensor electrodes were mounted (DataLITE, Biometrics Ltd., Ynysddu, UK, Table 2). GR and SR muscles pertained to brake operation, while GL and SL pertained to clutch operation. MVC is the highest muscle contraction which produces the highest-amplitude EMG signal and is treated as the reference for signal data processing and muscle fatigue assessment. The highest muscle contraction was induced through a strength measurement setup developed by the Central Institute of Agricultural Engineering, Bhopal, India (Figure 1a) [33] against maximum application force (592 ± 82 N by left leg and 632 ± 61 N by right leg) and MVC was measured in response.

The skin was inspected and sanitized, and then the EMG electrodes were placed under the supervision of a medical professional, in line with the selected muscles, to minimize the skin impedance and noise [8,9]. Electrodes were connected wirelessly to a remote computer to retrieve a continuous data stream in real-time in the provided software application. Three repeated measurements of 30,000 data points were collected for 30 s each for both the lower limbs during maximum muscle contraction measurements, while a total of 60,000 data points were collected for 60 s each during actual tractor clutch and brake pedal operations (Figure 1b). HR of the subjects was recorded using an HR monitor (Polar, Finland), and OCR was recorded using K4b2 portable metabolic analyzer (COSMED, Rome, Italy). Actuation forces of the brake and clutch were recorded using a custom-built instrumented foot transducer (IFT) [28,34]. The overall discomfort rating (ODR) of the subjects was recorded on a 10-point psychophysical rating scale (0: no discomfort [ND]; 3: light discomfort [LD]; 4: more than light discomfort [MLD]; 5: moderate discomfort [MD]; 6: more than moderate discomfort [MMD]; 7: uncomfortable [UC]; 10: extreme discomfort [ED]) which is an adoption of Corlett and Bishop technique [35]. Subjects were asked to rate their overall discomfort on a 10-point scale after each trial run.

These measurements were recorded for a total of 342 trials (19 operators × 3 tractors × 3 speeds × 2 surfaces) in a randomized order to minimize the error during data collection. The selected operational speeds were 4.2–4.3 km/h (S1), 4.3–4.7 km/h (S2), and 4.7–5.0 km/h (S3) and operating surfaces were tarmacadam and farm road surface (TR and FR). HR was recorded after the 6th minute of operation post-stabilization [36], and OCR was used to calculate EER Equation (1) [37].

EER = 20.93 × OCR(1)

### 2.3. Data Analysis

**Figure 2:**
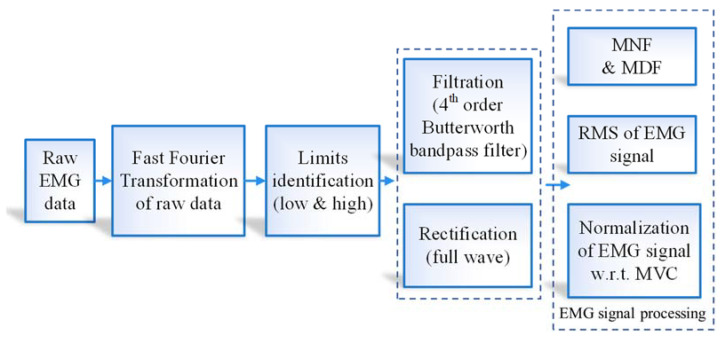
Flow chart for feature extraction of recorded EMG signals.

**Figure 3:**
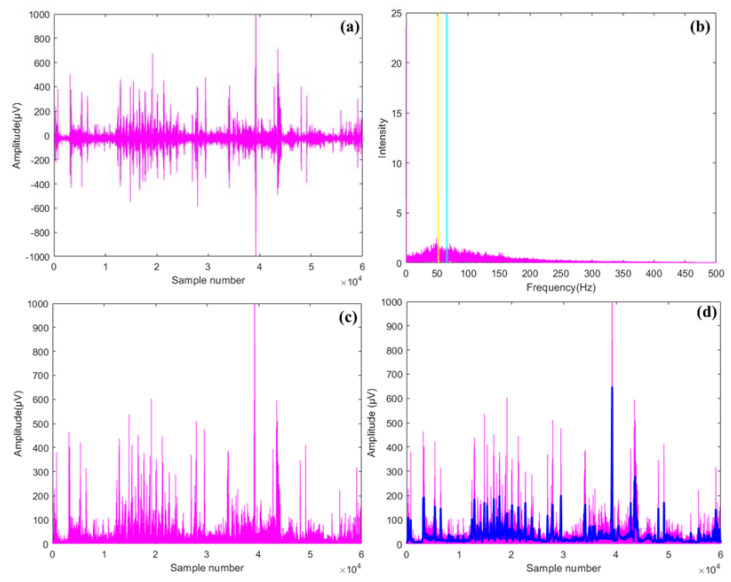
Electromyography derived (**a**) raw signal, (**b**) Fast Fourier Transformation (Cyan bar represents mean frequency [MNF] and Yellow bar represents median frequency [MDF]), (**c**) rectified signal, and (**d**) root mean square envelope in blue color.

**Figure 4:**
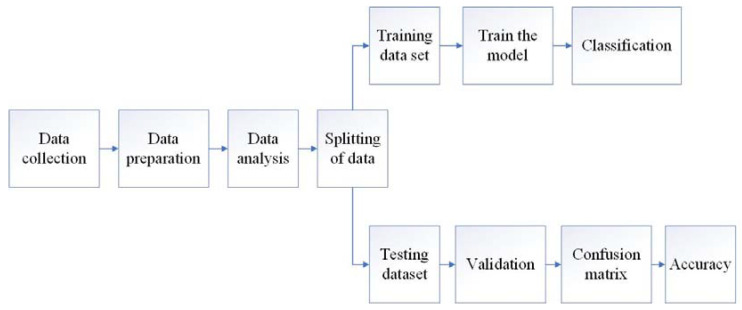
Flow chart for operator’s overall discomfort classification using a machine learning algorithm.

The raw EMG signals of the GR, GL, SR and SL muscles were preprocessed using MATLAB 2020b software (The MathWorks, Natick, MA, USA) using Fast Fourier Transform (FFT), rectification, filtration, and feature extraction (Figure 2, [4]). Firstly, the raw data was plotted in a time domain (Figure 3a) and transformed into a frequency domain through FFT along with the identification of mean and median frequencies (MNF, Equation (2), cyan bar in Figure 3b, and MDF, Equation (3), yellow bar in Figure 3b) for muscle fatigue assessments [38,39]. Next, data filtration was conducted using a fourth-order Butterworth filter and low- and high-frequency limits (20 and 300 Hz) [22]. Full wave rectification was then conducted to convert negative signals to positive ones for further analysis (Figure 3c). After filtration and rectification, root mean square (RMS, Equation (4)) [38] and muscle workload (%MVC, Equation (5)) [13] values were calculated for the respective muscles [40]. Figure 3d shows a sample RMS envelope for a muscle during operation. As a step towards identifying the factors affecting muscle fatigue and workload so that insights on workplace design reconsiderations and best operation practices can be derived, an N-way Analysis of Variance (ANOVA) was formulated (Python version 3.9). Operational speed, tractor model, and operating surface were selected as the independent variables, while RMS, %MVC, MNF, MDF, actuation force, HR, and EER were selected as the response variables. Pertaining to objective 2, Pearson correlations (normal data distribution) were assessed between RMS and EER, and RMS and actuation forces [14]. All statistical analyses were conducted at 5% significance (*p*-value = 0.05).

(2) $$\mathrm{MNF}=\sum_{j=1}^{M}P_{j}/M$$

(3) $$\mathrm{MDF}=\frac{1}{2}\sum_{j=1}^{M}P_{j}$$

(4) $$\mathrm{RMS}=\sqrt{\frac{1}{N}}\sum_{i=1}^{N}x_{i}{}^{2}$$

%MVC = RMS × 100/MVC(5)

where *Pj* = Power spectrum, *M* = power spectra length, *N* = number of samples, *i* = EMG data point.

### 2.4. Operator Workload Classification

In addition to manually recording ODR from the subjects, classification of overall discomfort was conducted (Figure 4) as the ODR can be subjectively intensified by numerous unaccounted psychological factors. For this, three best-data-size-suited machine learning algorithms were formulated: (1) k-nearest neighbor (KNN) with k value of 6, (2) random forest classifier (RFC) with n_estimators of 10, and (3) support vector machine (SVM) with a polynomial kernel. KNN is the simplest classifier that computes the distance of data points from the neighbors (k) and classifies them into different classes [41,42]. RFC uses a bagging approach to create several decision trees (n_estimators) where each node questions a datapoint, and the branches represent possible answers to that question [43]. SVM is a supervised machine learning classifier that classifies data into different domains by finding hyperplanes with a maximum margin [42]. RMS of electromyography (EMG-RMS), BMI, HR, and EER measurements were defined as the input variables during the recorded ODR ratings as the response variable. The response ODRs were also classified into six classes: ND, LD, MLD, MD, MMD, and UC classes, as mentioned in Section 2.2.

## 3. Results

### 3.1. Actuation Forces

**Figure 5:**
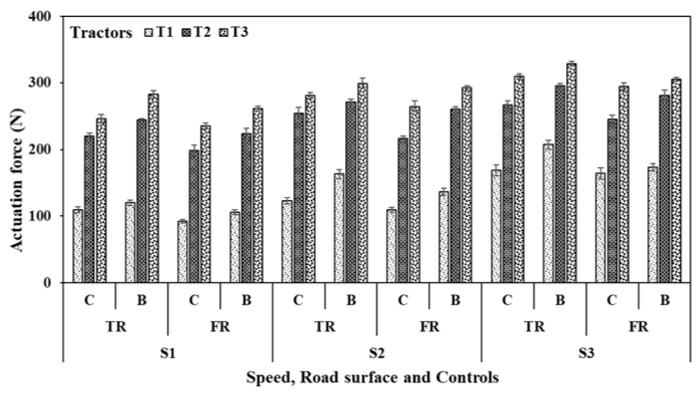
Actuation forces recorded during clutch and brake pedal operations of selected tractors. TR—Tarmacadam road surface, FR—Farm road surface; C—Clutch, B—Brakes. Further details are presented in Table 3.

At the selected operating speeds (S1–S3), clutch and brake actuation forces (Figure 5) were recorded in the ranges of 133 ± 28 N (mean ± standard deviation) and 163 ± 38 N, respectively, for tractor T1 on the tarmacadam road surface. Those observations on the farm surface were significantly lower and in the ranges of 122 ± 32 N and 138 ± 30 N, respectively. For tractor T2, the clutch and brake operation forces were much higher than tractor T1 and in the ranges of 247 ± 22 N and 270 ± 22 N, respectively, on the tarmacadam surface and 220 ± 20 N and 255 ± 25 N, respectively, on the farm surface. While for tractor T3, pertinent forces were further higher and in ranges of 279 ± 28 N and 304 ± 21 N, respectively, on the tarmacadam surface, and 265 ± 26 N and 287 ± 20 N on the farm surface. For all tractors, the actuation forces were significantly higher for brakes (Range: 120–305 N) compared to clutch (Range: 109–295 N, two sample *t*-tests, *p* < 0.01). The actuation forces for clutch and brake operations were significantly affected by the speed of operation, tractor type, operating surface, and their associated interactions (N-way ANOVA, *p* < 0.01). Additional details are summarized in Table 3.

### 3.2. Heart Rate (HR)

**Figure 6:**
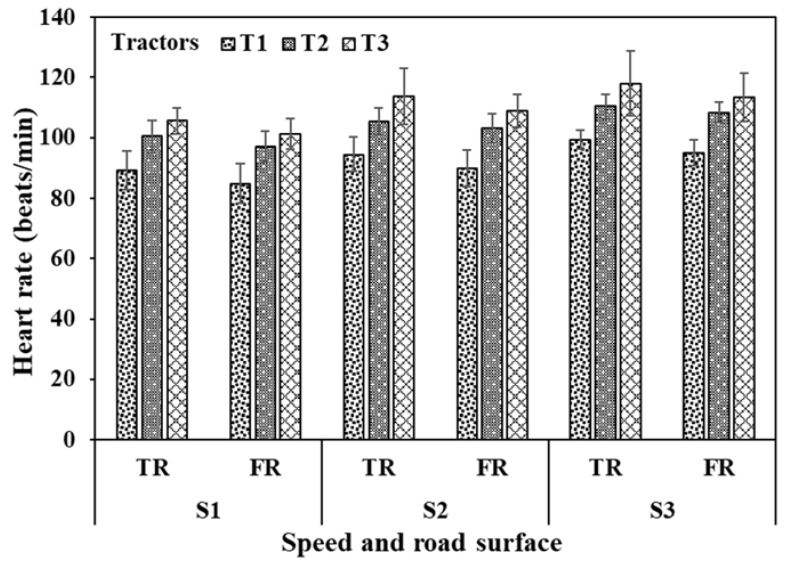
Heart rate of the operators during tractor clutch and brake operation. TR—Tarmacadam road surface, FR—Farm road surface; C—Clutch, B—Brakes. Further details are presented in Table 3.

The operator HR during the operation of tractor T1 ranged from 81–105 beats/min on the tarmacadam surface at all selected operating speeds (S1–S3), whereas on the farm surface, the values ranged from 80–100 beats/min. Such ranges for tractor T2 were higher, i.e., 95–117 beats/min and 94–115 beats/min, respectively. For tractor T3, the values were even higher and ranged between 100–131 beats/min on the tarmacadam surface and 99–125 beats/min on the farm surface (Figure 6). HR was recorded to be higher for operations on tarmacadam roads (90–118 beats/min) compared to the farm road (85–113 beats/min). ANOVA revealed that HR was significantly affected by the operation speed, tractor type, and road surface (*p* < 0.01). HR of operators were observed in the light (<90 beats/min) to heavy workload ranges (110–130 beats/min) [44]. Additional details are summarized in Table 3.

### 3.3. Energy Expenditure Rate

**Figure 7:**
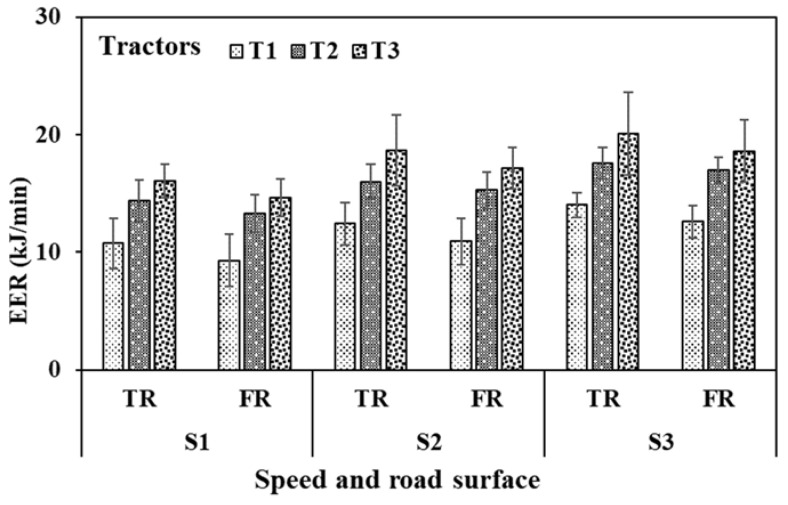
Energy expenditure rate of the subjects during tractor clutch and brake operation. TR—Tarmacadam road surface, FR—Farm road surface; C—Clutch, B—Brakes. Further details are presented in Table 3.

The EER of the operators for operating tractor T1 ranged between 9–15 kJ/min on the tarmacadam surface and 7–13 kJ/min on the farm surface at selected operating speeds. Such ranges for tractor T2 were higher, i.e., 13–19 kJ/min on the tarmacadam surface and 11–17 kJ/min on the farm surface. While for tractor T3 those ranges were further higher, i.e., 14–24 kJ/min on the tarmacadam surface and 13–21 kJ/min on the farm surface (Figure 7, additional details are presented in Table 3). EER was affected by the operation speed, tractor type and road surface (*p* < 0.01). Some EER observations were in the light load (<9 kJ/min) but mostly within the moderate to heavy load categories (18–27 kJ/min) [45].

### 3.4. Electromyography Features

#### 3.4.1. Root Mean Square (RMS) and Muscle Workload (%MVC)

**Figure 8:**
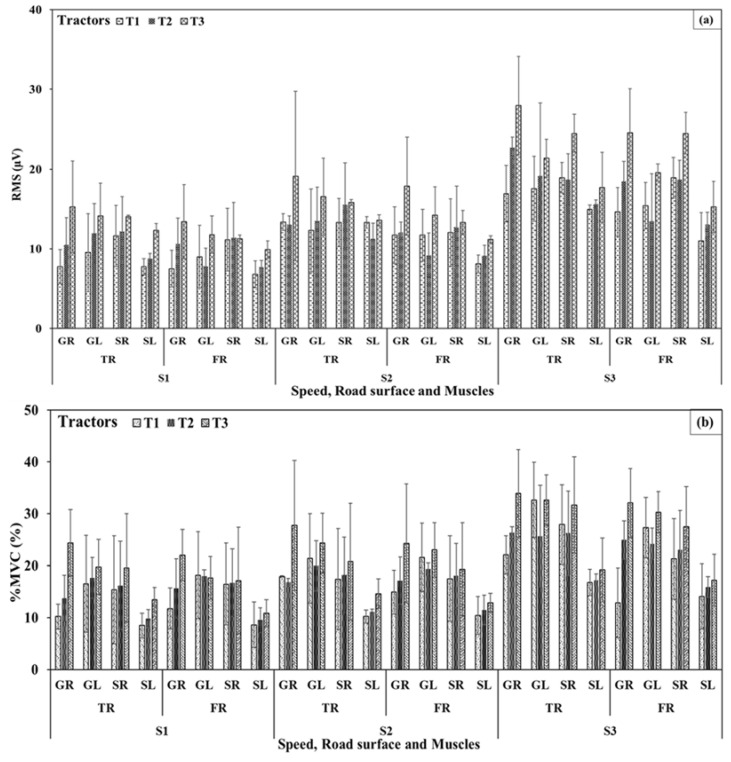
Electromyography (**a**) root mean square and (**b**) %maximum volumetric contraction. TR—Tarmacadam road surface, FR—Farm road surface; C—Clutch, B—Brakes. Further details are presented in Table 3.

At selected speeds, EMG-RMS (Figure 8a) of the GR and SR muscles (involved in brake operation) were in the ranges of 13 ± 4, 15 ± 6, 21 ± 9 µV and 14 ± 4, 16 ± 5, and 18 ± 5 µV for T1, T2, and T3 tractors, respectively, on the tarmacadam surface. Those observations on the farm surface were lower and recorded as 11 ± 3, 14 ± 4, 19 ± 8 µV and 13 ± 4, 14 ± 5, and 15 ± 5 µV. RMS for GL and SL muscles (involved in clutch operation) were obtained in the ranges of 13 ± 5, 15 ± 6, 17 ± 5 µV and 11 ± 3, 12 ± 3, 15 ± 3 µV for T1, T2, AND T3 tractors on the tarmacadam surface. Such observations on the farm surface were lower and in the ranges of 12 ± 4, 10 ± 4, 15 ± 4 µV and 9 ± 3, 10 ± 3, and 12 ± 3 µV on the farm surface. EMG-RMS for all the muscles were affected by speed, tractor type and operating surface (*p* < 0.01). Additional details are presented in Table 3.

%MVC (Figure 8b) of the GR and SR muscles (brake operation) for tractors T1, T2, AND T3 were observed in the ranges of 17 ± 5, 19 ± 6, 26 ± 10% and 20 ± 10, 21 ± 8, 24 ± 10%, respectively, on the tarmacadam surface and 15 ± 5, 19 ± 6, 24 ± 9% and 18 ± 7, 19 ± 6, 21 ± 10%, respectively, on the farm surface at selected speeds. %MVC of the GL and SL muscles (clutch operation) were observed in the ranges of 21 ± 8, 22 ± 7, 28 ± 6% and 11 ± 4, 13 ± 3, 16 ± 4% on the tarmacadam surface while, 22 ± 7, 22 ± 3, 26 ± 5% and 11 ± 5, 12 ± 3, 14 ± 4% on the farm surface for tractors T1, T2, and T3 at selected speeds. %MVC for all the muscles were affected by the speed and tractor type (*p* < 0.01) but not the operating surface. Muscle activation (%MVC) was higher for tractor T3 followed by tractor T2 and T1. Additional details are presented in Table 3.

#### 3.4.2. Mean and Median Frequency

**Figure 9:**
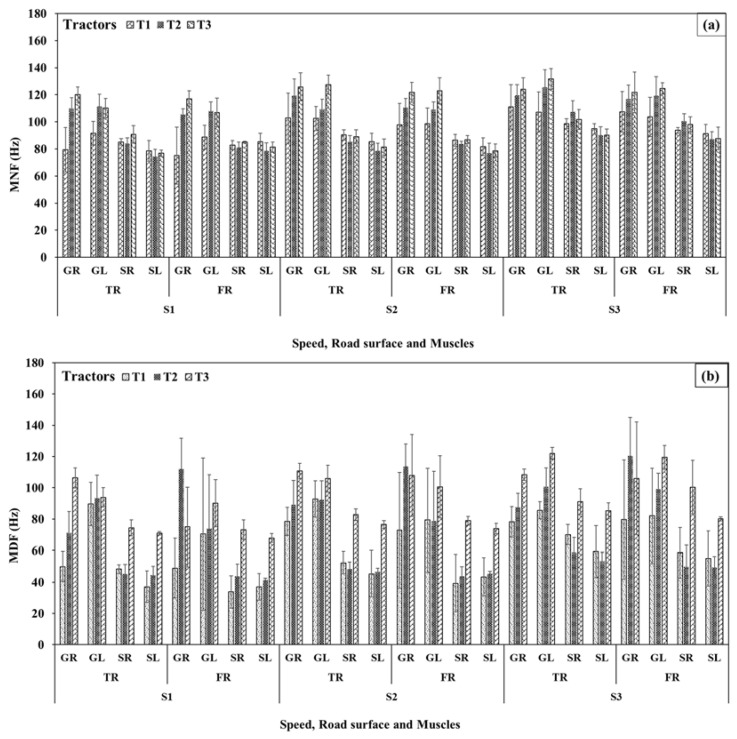
Electromyography (**a**) mean, and (**b**) median frequencies of evaluated muscles during tractor brakes and clutch operations. TR—Tarmacadam road surface, FR—Farm road surface; C—Clutch, B—Brakes. Further details are presented in Table 3.

At the selected forward speeds, MNF of the GR and SR muscles (brake operation) were observed in the ranges of 98 ± 20, 116 ± 10, 123 ± 8 Hz and 91 ± 7, 92 ± 12, 94 ± 8 Hz on the tarmacadam surface for tractors T1, T2, and T3, respectively (Figure 9a). Those observations on the farm surface were lower and in the ranges of 93 ± 21, 111 ± 8, 120 ± 7 Hz and 87 ± 5, 88 ± 10, and 90 ± 7 Hz. Pertinent values for GL and SL were recorded in the ranges of 100 ± 13, 115 ± 10, 123 ± 12 Hz and 86 ± 9, 81 ± 9, and 83 ± 7 Hz for T1, T2, and T3 tractors on the tarmacadam surface, and 97 ± 12, 112 ± 5, 118 ± 11 Hz and 83 ± 9, 79 ± 8, and 80 ± 8 Hz, respectively, on the farm surface.

MDF (Figure 9b) of the GR and SR muscles for tractors T1, T2, and T3 were observed in the ranges of 68 ± 16, 82 ± 14, 108 ± 5 Hz and 57 ± 11, 51 ± 8, and 83 ± 9 Hz on the tarmacadam surface, and 67 ± 31, 115 ± 18, 96 ± 29 Hz, 44 ± 17, 46 ± 9, and 84 ± 15 Hz, respectively, on the farm surface. Pertinent observations for GL and SL were in the ranges of 89 ± 10, 95 ± 12, 107 ± 13 Hz and 47 ± 15, 48 ± 5, 78 ± 7 Hz for tractors T1, T2, and T3, respectively, on the tarmacadam surface, and 77 ± 33, 84 ± 27, 103 ± 18 Hz and 45 ± 14, 45 ± 5, 74 ± 6 Hz, respectively, on the farm surface. Additional details are presented in Table 3. RMS, %MVC, MDF, and MNF as the indicators of muscle activation, workload, and strain were higher for tractor T3, followed by T2 and T1. The values were also higher for operations on the tarmacadam surface compared to the farm road.

### 3.5. Relationships between Actuation Forces, Energy Expenditure Rate, and Electromyography

**Figure 10:**
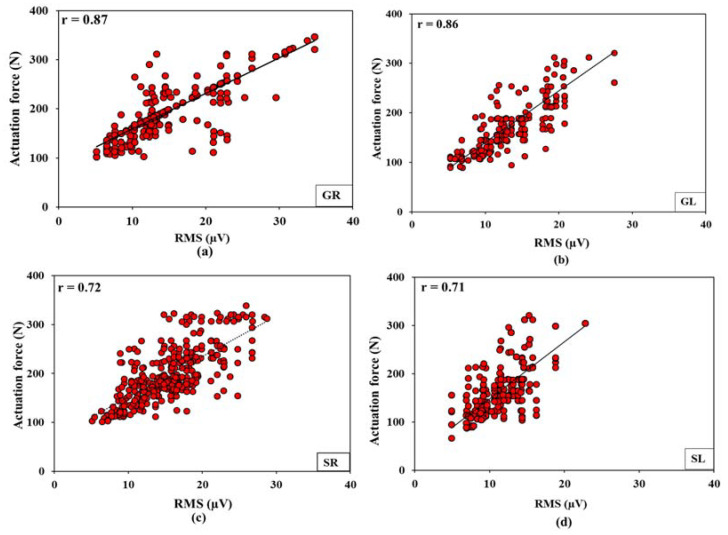
Correlations between actuation forces and electromyography feature (RMS) (**a**) gastrocnemius right, (**b**) gastrocnemius left, (**c**) soleus right, and (**d**) soleus left muscles. Right muscles: brake operation, left muscles: clutch operation.

**Figure 11:**
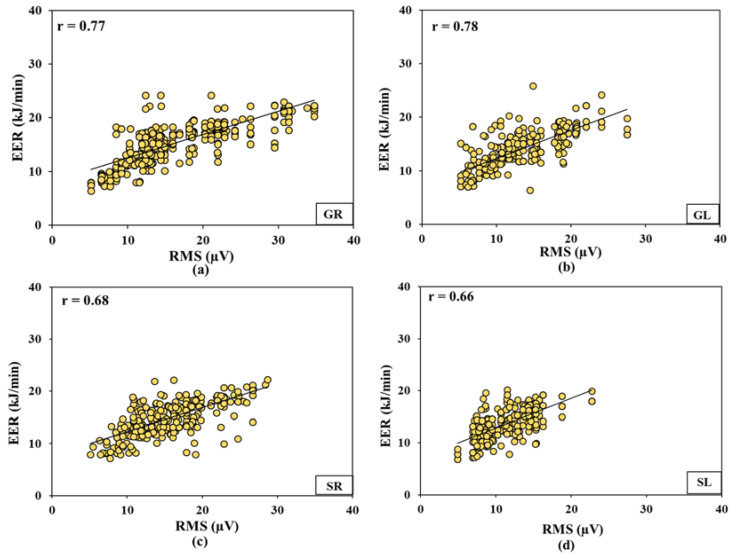
Correlation between electromyography feature (RMS) and energy expenditure rate for (**a**) gastrocnemius right, (**b**) gastrocnemius left, (**c**) soleus right, and (**d**) soleus left muscles. Right muscles: brake operation, left muscles: clutch operation.

The clutch and brake actuation forces were observed to have a positive-significant and moderate-high correlation with EMG-RMS of the selected muscles (***r***: 0.71–0.87, *p* < 0.01, Figure 10). EER for brake operations had a relatively higher correlation with the EMG-RMS of gastrocnemius muscles (***r***_GR_ = 0.77, ***r***_GL_ = 0.78) compared to soleus muscles (***r***_SR_ = 0.68, ***r***_SL_ = 0.66, Figure 11). Higher correlations for GR and GL muscles could be mostly because those muscles are responsible for knee flexion-extension and foot plantar dorsiflexion.

### 3.6. Overall Discomfort Rating (ODR) and Its Classification

**Figure 12:**
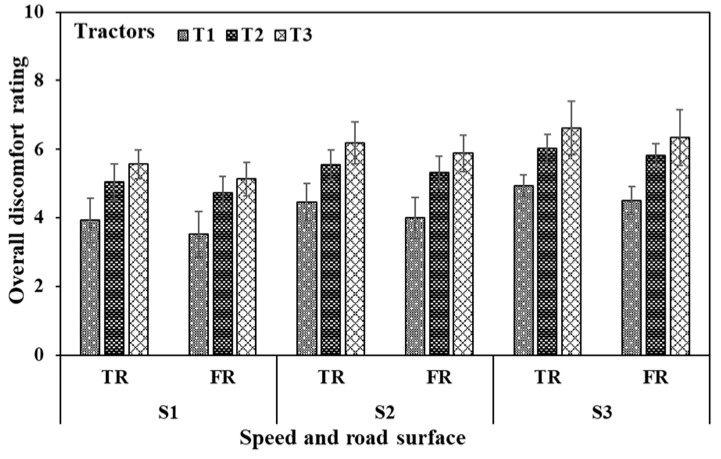
Overall discomfort rating of operators during the tractor clutch and brake operations.

**Figure 13:**
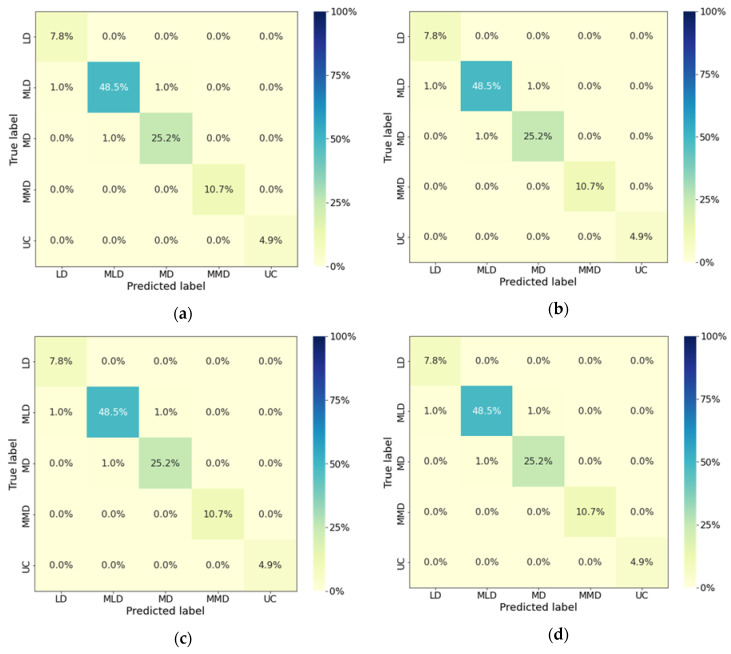
Random forest classified muscle workload for (**a**) gastrocnemius right, (**b**) gastrocnemius left, (**c**) soleus right, (**d**) soleus left. Right muscles: brake operation, left muscles: clutch operation. LD—light discomfort, MLD—more than light discomfort, MD: moderate discomfort, MMD—more than moderate discomfort, UC—uncomfortable.

Post-operation trials, operator ODR (Figure 12) responses were in the ranges of 3.2–5.7 (LD to MMD) for the T1 tractor at all the operating speeds on the tarmacadam surface and 3.0–5.3 on the farm road surface (LD to MD). For the T2 tractor, ODR ranged within 4.5–6.7 (MLD to UC) on the tarmacadam road surface and within 4.3–6.4 (MLD to UC) on farm road surface for all selected operation speeds. For the T3 tractor, the values were in the ranges of 5.1–7.8 (MD to below ED), and 4.9–7.4 (MD to below ED) on tarmacadam and farm road surface, respectively. ODR Observations determine that tractor operation on the tarmacadam surface was more strenuous than the farm surface (Table 3). Furthermore, the operation with T3 induced more workload, followed by that from T2, and T1. When evaluated statistically, ODR was observed to be affected by the operation speed, tractor type and road surface (*p* < 0.01). When ODR was classified for the workload on all selected muscles, maximum accuracy was yielded by RFC machine learning algorithm (97%, Table 4), followed by SVM (92–96%) and KNN (87–91%). RFC classified 7.8% operations in LD, 48.5% in MLD, 25.2% in MD, 10.7% in MMD, and 4.9% in UC categories, and misclassified 2% operations in MLD and 1% operations in MD category (Figure 13).

## 4. Discussion

Clutch and brake actuation forces were at maximum for tractor T3 where both are mechanically actuated and therefore require larger muscle work for operation. Secondly, in case of mechanical controls, internal frictions increase wear and tear as the operation age progresses. These observations are well supported by the findings concluded in the literature that nominally fixed machine joints are subjected to micro-mobility followed by wear due to vibrations. Extra forces and muscular actions are required to counter these vibrations [46]. In the case of relatively newer tractors (T1 and T2), upgradations of hydraulically operating brakes and clutches have been included. These upgrades reduce the vibrations and internal frictions, therefore, the magnitude of reactive and required actuation forces [46]. Similar observations of higher clutch actuation forces were reported by Fallahi et al. [32], where the older tractor (75 HP) required a higher clutch actuating force of 340 N compared to the newer and upgraded tractor (100 HP) that required 290 N of force, despite the former tractor being of lower power capacity. A study on a range of passenger vehicles [47] also documented that the mean braking distance increased by 22%, deacceleration reduced by 13%, and braking time to reduce the speed by 16 km/h increased by 27% when manual brake systems were used relative to hydraulically actuated brakes. Clutch and brake actuation forces increased with the increased speed of operation. This can be explained by a well-established fact that higher force is required to break the motion when moving at higher speeds or accelerations [48,49]. A study by Mortimer et al. [47] also reported that the deacceleration of the vehicle traveling at 56 km/h was higher than the vehicle travelling at 80 km/h and concluded that it was more difficult for the subjects to stop a faster-moving vehicle. The study also reported higher braking distances and time against higher speeds. The reason for brake and clutch actuation forces to be higher on the tarmacadam surface compared to the farm surface is that the tarmacadam road has a relatively lower coefficient of rolling friction and, therefore, a higher slipping potential where braking a vehicle would require larger actuation forces [50]. This is also observed in the prior studies that reported higher braking distances and time required for a vehicle moving on wet/slippery surfaces compared to dry surfaces [47,51].

Clutch and brake actuation forces also conform with the EMG responses. Higher forward speeds lead to higher RMS, %MVC, MNF, and MDF values and vice-versa at lower forward speeds for all the evaluated muscles. These observations are due to increased motor unit action potentials (recorded by EMG), muscle contraction intensity, speed of activation potential, and a number of active motor neurons that increased in direct proportion with the muscle engagement against required actuation forces [14,52]. This was very well evident from the moderate to moderately-high correlations observed between the RMS values for all the involved muscles and actuation forces (***r***_GR_ = 0.77, ***r***_GL_ = 0.78, ***r***_SR_ = 0.68, ***r***_SL_ = 0.66, Figure 11). Therefore, similar reasons that stand for the highest actuation forces for tractor T3, followed by T2 and T1, remain valid for higher EMG signal response orders for those tractors, operating surfaces, and speeds [53]. Such responses are further complemented by a prior study on tractor clutch actuation forces [32], where the pain threshold reduction for GL muscle within 30–60 s of actuation was higher pertaining to the older tractor with mechanical clutch (3.87–6.30 N) compared to a newer tractor with upgraded clutch mechanism (3.23–4.30 N). The study also reported that the pain threshold point was hit much earlier in the case of the older tractor. RMS, MNF, MDF, and %MVC for GL muscle were higher than GR muscle for tractors T1 and T2 but were lower for T3 (Table 3) as the former tractors have mechanically actuated clutches and hydraulically actuated brakes, while T3 has both mechanically actuated clutch and brakes. %MVC was higher for the GL muscle compared to the GR muscle, possibly because the frequency of clutch operation is higher than the brake operation. In the case of the lower limbs, the GL muscle is the most affected muscle among tractor operators because it is involved in planter flexion of ankle that is engaged in clutching; this finding has also been reported by Fallahi et al. [30]. Nonetheless, EMG signal responses clearly outline that muscle fatigue, operation workload, and occupation risk can be higher for older tractors with mechanically actuated controls, at higher operating speeds, and over smoother surfaces. This is evident from the %MVC for GR, GL, and SR muscles that were beyond the recommended limit (30%) for tractor T3 at speed S3.

The MNF and MDF signals also represent muscle fatigue and are affected by the operation durations and muscle fiber composition and distribution in individual subjects [4,15,16,54,55]. Higher values of MDF and MNF are clearly observable from Table 3, where the values were higher for tarmacadam surface, higher speeds, and tractors that mechanically actuated controls indicative of the heavy engagement for larger/prolonged duration. Similar observations were reported by Phinyomark et al. [55] where the amount and duration of muscle forces proportionally impacted MNF and MDF values in addition to the subject-dependent-anthropometric characteristics, muscle fiber types and dimensions. ODR ratings which were lowest for tractor T1, followed by tractors T2 and T3, also conformed with the EMG and forced responses of muscle strain and workloads. ODRs were also higher for higher forward speeds as those resulted in increased vibrations and enhanced muscle activities [56].

Similar to actuation force, EMG and ODR responses and HR and EER observations were also the highest for the tractor T3, tarmacadam surface, and highest speed (S3) combination followed by for other combinations of the independent factors. This was well represented in their correlation plots with RMS values that ranged between moderate to moderate-high (Figure 11). Similar observations of these biomechanical indicators were reported in prior studies where vibrations at higher speeds increased HR and EER [57,58]. This is because of the action potentials of the muscle fiber beneath the skin that increases with the muscle firing rates as a result of the increased actuation forces [58]. Specifically, HR and EER relate to muscle contractions that occur in four different phases: (1) Adenosine triphosphate (ATP) hydrolysis that reorients and energizes the myosin, (2) attachment of myosin to actin to form cross-bridges, (3) power stroke with cross-bridge rotation towards the sarcomere’s center, and (4) detachment of myosin from actin after the power stroke [4]. Significantly high correlations observed between actuation forces and muscle-specific EMG responses as well as between EER and muscle-specific EMG responses, suggest that it might not always be necessary to use EMG sensors, which require mounting electrodes on the skin surface—using simpler ergonomic transducers can also yield the muscle fatigue and workload assessments under resource-constrained situations. Such situations may not be common to the automobile, aviation, or other production engineering sectors but may be very common to the agricultural mechanization sector that encompasses a range of operation types apart from the tractor-based, as well as varied socio-economic backgrounds mostly visible in the developing countries.

ODR can often become subjective, and to minimize that, RFC categorization of ODR at sufficiently high accuracies may be helpful. Utilization of multiple automated responses from other independent ergonomic transducers, i.e., BMI, HR, EER and EMG-RMS, also aided in minimizing subjectivity and enhancing accuracy. Earlier studies have determined overall workload classes solely based on RMS responses and obtained slightly lower or similar accuracies using RFC machine learning [43,59]. RFC performed well compared to other machine learning algorithms (Table 4) because it uses a bagging approach to create a bunch of decision trees with a random subset of the data and then trains the model several times on a random sample to achieve good prediction performance.

## 5. Conclusions

Findings of the study in real conditions and with real operators clearly outline it is possible to quantify lower limb muscle workload and fatigue using high-throughput ergonomic transducers. Along these lines, the GL and SL muscles experienced higher strain resulting from higher clutch actuation forces (90–312 N) compared to the brakes (105–332 N) that used the GR and SR muscles. The fatigue and workloads were assessed using EMG, force transducers, and other biomechanical measurements aggravated at the highest selected operating speed, tarmacadam surface, and for the oldest of the tractors that had mechanically actuated clutch and brake controls. The aggravation was such that it exceeded the allowable limits of muscle contraction (i.e., of 30% where GR: 43%, SR: 41%, GL: 38%). HR and EER were recorded in moderately heavy to heavy categories, which increased with the operation speed and were highest for the oldest of all the tractors (100–132 beats/min, 15–20 kJ/min). Well supported by the ODR responses in moderate to uncomfortable categories, observations of the study suggest a critical reconsideration of the designs of existing workplaces with tractors.

Clutch and brake actuation forces and EER mostly held significantly high correlations (***r***: 0.68–0.87) with the muscle-specific activity responses, suggesting that a comprehensive assessment of muscle workload and fatigue can also be conducted using simpler force measuring and ergonomic transducers. Machine learning showed the possibility of classifying operator discomfort at maximum accuracy (up to 97%).

Amid the limited, documented assessment of muscle workload and fatigue resulting from lower limb-based actuation of tractor clutch and brake systems in real conditions, the findings of the study are critical. This study, although focused on operators and tractors in Indian conditions, could be very well adopted on a global scale. More studies are required to efficiently model and simulate muscle workload, fatigue, and overall discomfort from tractor or tractor-driven machinery operations. Eventually, modeled and simulated findings assist in the development of a global protocol for designing efficient and safe operator workplaces or reconsidering the designs of existing tractor workplaces. This is critically needed to enhance work efficiency and agricultural productivity as agricultural mechanization advances globally.

## Figures and Tables

**Table 1:** Specifications of the selected tractors in the study.

Parameter (unit)	Tractors		
	T1	T2	T3
Make and Model	New Holland 6010	John Deere 5055E	Escort 355
Power (HP)	60	55	47
Rated rpm	2200	2400	2100
Forward speeds	12	9	10
Reverse speeds	12	3	2
Clutch type	Mechanical	Mechanical	Mechanical
Brake type	Hydraulic, oil-immersed multi-disc	Self-adjusting, self-equalizing, oil-immersed disc	Mechanical
Operational age	>3 years	>10 years	>15 years
Mass (kg)	2415	2110	1760
Tire condition	Good, negligible wear and tear	Slightly worn out	Slightly worn out
Front tire size Rear tire size	7.5 × 16 16.9 × 28	6.5 × 20 16.9 × 28	6.0 × 16 14.9 × 28

**Table 2:** Sensor and instrumentation specifications used in the study.

Instruments	Specifications	Measurement
Data LITE Surface EMG sensor (Biometrics Ltd., UK)	Integral dry reusable, Bandwidth: 10–250, 470, 950; Full scale: +/− 6 mV Peak to Peak; Gain: +/− 60 mV to +/− 6000 mV; Accuracy: +/− 1.0%	EMG signal response of muscles
Instrumented foot transducer (IFT)	Accuracy: +/− 0.1%	Actuation force of clutch and brake pedals
Heart rate monitor (Polar, Finland),	Operating temp: −10 °C to +50 °C Accuracy: +/− 0.01%	Heart rate of the operators
K4b2 portable metabolic analyzer	Accuracy: +/− 0.02%	Oxygen uptake of the operators

**Table 3:** Mean of physiological and electromyography responses measured for the selected subjects pertinent to clutch and brake operations with three tractors at three operating speeds and on two operating surfaces.

		Tarmacadam Surface									Farm Road								
Muscle/Leg	Response	T1			T2			T3			T1			T2			T3		
		S1	S2	S3	S1	S2	S3	S1	S2	S3	S1	S2	S3	S1	S2	S3	S1	S2	S3
**RL**	**AF**	120	163	207	245	271	296	283	299	329	106	136	173	224	261	281	262	292	305
**LL**	**AF**	109	123	169	220	254	267	246	281	310	92	109	164	198	217	246	236	265	295
**Full body**	**HR**	90	95	99	100	105	110	106	114	118	85	90	95	97	103	108	101	109	113
**Full body**	**EER**	11	12	14	14	16	18	16	19	20	9	11	12	13	15	17	15	17	19
**Full body**	**ODR**	3.9	4.5	4.9	5.0	5.5	6.0	5.5	6.2	6.6	3.5	4.0	4.5	4.7	5.3	5.8	5.1	5.9	6.3
**GR**	**RMS**	8	13	17	11	13	23	15	19	28	8	12	15	11	12	19	13	18	25
	**MNF**	79	103	111	110	119	119	120	126	124	75	98	107	105	110	117	117	121	122
	**MDF**	50	79	78	71	89	87	106	111	108	49	73	80	112	114	121	75	111	108
	**%MVC**	10	18	22	13	17	26	24	28	34	11	15	13	16	17	25	22	24	32
**GL**	**RMS**	10	12	18	12	14	19	14	17	21	9	12	15	8	9	14	12	14	20
	**MNF**	92	102	107	111	110	126	110	128	132	89	99	104	108	109	119	107	123	125
	**MDF**	90	93	86	93	92	101	94	106	122	71	79	82	74	79	99	90	101	120
	**%MVC**	16	21	33	17	20	26	20	24	33	18	22	28	18	19	24	18	23	30
**SR**	**RMS**	12	13	19	12	16	19	14	16	25	11	12	19	11	13	19	11	13	25
	**MNF**	85	90	99	89	85	107	91	89	102	83	86	9	81	84	101	85	87	98
	**MDF**	48	52	70	45	48	59	74	83	91	34	39	59	44	44	50	73	79	100
	**%MVC**	15	17	28	16	18	26	19	21	32	16	17	21	17	18	23	17	19	28
**SL**	**RMS**	8	13	15	9	11	16	12	14	18	7	8	11	8	9	13	10	11	15
	**MNF**	79	85	95	74	79	90	77	81	90	76	82	91	74	77	87	75	79	88
	**MDF**	37	45	59	45	47	53	71	77	85	37	43	55	41	46	49	68	74	80
	**%MVC**	8	10	17	9	11	17	13	15	19	8	10	14	9	11	16	11	13	17

RL—Right leg, LL—Left leg, S1, S2, S3 represent three speed ranges described in Section 2.3, T1, T2, and T3 are the tractors, GR—gastrocnemius right, GL—gastrocnemius left, SR—soleus right, SL—soleus left muscles. AF—Actuation force (N), HR—Heart rate (beats/min), EER—Energy expenditure rate (kJ/min), ODR—Overall discomfort rating, RMS—Root mean square of electromyography (µV), MNF—Mean frequency of electromyography (Hz), MDF—Median frequency of electromyography (Hz). MVC—Maximum volumetric contraction of electromyography (%). It must be noted that RL, GR, and SR pertained to brake operations and LL, GL, and SL pertained to clutch operations.

**Table 4:** Accuracy of operator’s overall discomfort classification using machine learning algorithms.

Accuracy (%)				
Classifier	GR	GL	SR	SL
KNN	91	87	90	91
RFC	97	97	97	97
SVM	96	92	93	96

KNN—K-nearest neighbor, SVM—Support Vector Machine, RFC—Random Forest classifier.

## Data Availability

Data will be provided on request.
